# Beyond structural domains: the emerging roles of PDLIM2 in cellular signaling and cancer progression

**DOI:** 10.3389/fphys.2025.1569285

**Published:** 2025-05-22

**Authors:** Yulian Shi, Tingyu Fan, Yuchao Yang, Jiaxuan Liu, Jun Ouyang, Jingxing Dai

**Affiliations:** ^1^ National Key Discipline of Human Anatomy, School of Basic Medical Sciences, Southern Medical University, Guangzhou, China; ^2^ Guangdong Provincial Key Laboratory of Digital Medicine and Biomechanics, Guangzhou, China; ^3^ Guangdong Engineering Research Center for Translation of Medical 3D Printing Application, Guangzhou, China; ^4^ National Virtual & Reality Experimental Education Center for Medical Morphology (Southern Medical University), Guangzhou, China

**Keywords:** PDZ and LIM domain 2 (PDLIM2), cytoskeleton-related protein, nuclear factor kappa-B (NF-κB), cell polarization, cytoskeleton

## Abstract

The cytoskeleton not only provides structural support for cells but also plays a crucial role in intracellular information transmission. Cytoskeleton-associated proteins are intricately involved in, and indispensable for, regulating cytoskeletal dynamics. PDLIM2, also known as mystique or SLIM, is localized in the nucleus and cytoplasm and functions as a cytoskeleton-associated protein that facilitates binding of other proteins to the cytoskeleton. PDLIM2 exhibits widespread expression in various tissues and cell types, contributing to cellular proliferation and differentiation processes. This review provides a concise overview of PDLIM2, including its genetic background, structural features, involvement in tumorigenesis and development, as well as potential molecular signaling pathways. Lastly, we address the current limitations in PDLIM2 research while highlighting future prospects.

## 1 Introduction

In humans, the PDZ and LIM domain protein (PDLIM) family consists of seven members (i.e., PDLIM1-PDLIM7) (Healy and Collins, 2023; Huang et al., 2020). The PDLIM family is closely associated with cytoskeletal actin (Huang et al., 2021), serving as adaptor proteins for protein-protein interactions and facilitating interactions between other proteins and the cytoskeleton to exert specific biological effects, including stress fiber repair. PDLIM proteins are closely linked to cell proliferation, differentiation, migration, cell polarity, and cell-cell adhesion (Guo and Qu, 2021; Yang Y. et al., 2024). This review particularly focuses on the role of PDLIM2 (also known as mystique or SLIM), which is implicated in membrane transport and cellular movement (Torrado et al., 2004; Yuk et al., 2021). PDLIM2 interacts with actin and myosin while also binding to α-actinin and myosin heavy chain, and other actinins. Moreover, PDLIM2 is localized within the actin cytoskeleton network, where it plays a significant role in epithelial cell migration. Additionally, PDLIM2 activity is associated with nuclear factor kappa-B (NF-κB), signal transducer and activator of transcription (STAT) as well as β-catenin (Guo and Qu, 2021). Importantly, dysregulation of PDLIM2 can have severe consequences, including cancer. Because in our experiment we found that the expression of PDLIM2 was significantly elevated during the osteogenic differentiation process of cells, we wanted to understand the PDLIM2 gene, its expression and the involved signaling pathways. This review will discuss various aspects including the gene expression profile of PDLIM2, along with its structure distribution patterns and its involvement in tumor development signaling pathways.

## 2 *PDLIM2* gene structure, protein domains, and tissue distribution

The human *PDLIM2* gene is located on chromosome 8p21, while mouse mystique shares homology with human mystique2. However, these two cDNAs differ due to the absence of a 500 bp 5′-UTR (Loughran et al., 2005). PDLIM2 belongs to the actin-related LIM protein family (Torrado et al., 2004), comprising an amino-terminal PDZ domain and a carboxy-terminal LIM domain. The PDZ domain, consisting of approximately 80–100 amino acids, facilitates specific protein-protein interactions and plays a crucial role in protein complex assembly. Proteins containing the PDZ domain are implicated in intracellular signaling (Torrado et al., 2004; Ponting et al., 1997). The LIM domain is characterized by its zinc finger structure, which is rich in cysteine residues (Michelsen et al., 1993) and spans about 50–65 amino acids in length (Schmeichel and Beckerle, 1994). Proteins harboring a LIM domain play significant roles in cytoskeletal tissue function and are also associated with tumorigenesis and tumor progression (Bach, 2000). PDLIM2 exhibits differential distribution across various tissues, with the highest expression levels observed in the heart and moderate expression levels detected in the spleen, kidneys, and testes. The lowest PDLIM2 expression has been found in the brain (Loughran et al., 2005).

## 3 The role of PDLIM2 in cell polarization

PDLIM2 is essential to produce healthy polarized human mammary epithelial cell acini (Deevi et al., 2014). Polarization refers to the differentiation of distinct inter- and extracellular regions, leading to the development of diverse characteristics and functions inside and outside the acinar structure in normal breast epithelial cells. The polarization process is crucial for proper breast development and function in normal mammary epithelial cell acini. PDLIM2 plays a significant regulatory role in the integrin β1 signaling pathway involved in breast epithelial polarization and acinar formation. PDLIM2 governs integrin β1-mediated signaling pathways, and its inhibition results in hyperactivation of this pathway, leading to aberrant polarization, proliferation/apoptosis imbalance, dysregulation of cellular matrix adhesion, as well as disruption of acinar formation in normal human mammary epithelial cells. Importantly, in one study, the authors also found that PDLIM2 regulates the integrin β1-RhoA signaling pathway. It plays an important role in cell polarization of cultured cells *in vitro*. Inhibition of FAK or ROCK reversed migration defects caused by PDLIM2 loss, suggesting that PDLIM2 may promote cell contraction and movement by activating these kinases (Deevi et al., 2014).

Moreover, PDLIM2 is commonly associated with M2 macrophages in breast cancer and is required for their migration. PDLIM2 loss hindered M2 polarization and led to decreased expression of CD206^+^ and YM-1. High PDLIM2 expression is associated with M2 macrophage infiltration, which is often considered a hallmark feature of invasive breast cancer, particularly triple-negative breast cancer (Cox et al., 2022). Triple-negative breast cancer does not express the estrogen receptor, progesterone receptor, and human epidermal growth factor receptor-2 (HER-2), thereby limiting the options for targeted therapies. Therefore, investigating the potential of targeted drugs to downregulate PDLIM2 expression, specifically in triple-negative breast cancer patients, may aid the development of future therapeutic strategies against this cancer subtype. However, further studies are required to validate this hypothesis (Cox et al., 2022; Qu et al., 2010a).

## 4 PDLIM2-associated signaling pathways

### 4.1 PDLIM2 and NF-κB signaling pathway

NF-κB is a classical pathway in the inflammatory process and under normal circumstances, this pathway is strictly regulated. Typically, Members of the NF-κB family (such as p50 and p65) form dimers (heterodimers or homodimers) in the cytoplasm and associate with inhibitory protein IκB (such as IκBα) to form trimers (p50/p65/IκBα). IκB binds to the nuclear localization sequence (NLS) of NF-κB and prevents it from entering the nucleus, thereby keeping it in an inactive state. When cells are stimulated by external factors such as LPS, TNF-α or IL-1, these signals trigger a cascade reaction by activating the IKK complex. The activated IKK complex mediates the phosphorylation modification of IκBα, which in turn leads to its ubiquitination marking and ultimately results in the degradation of IκBα by the proteasome. This process releases the originally bound NF-κB dimer (such as p50/p65), exposing its nuclear localization sequence (NLS). The exposed NLS guides the NF-κB dimer to transport through the nuclear pore complex into the nucleus, and subsequently specifically binds to the promoter regions of target genes (such as IL-6, TNF-α), initiating the transcriptional expression of inflammatory mediators. Therefore, long-term or continuous activation of NF-κB is not conducive to human health, as this can lead to the onset of inflammatory diseases (e.g., certain types of sclerodermas and asthma, among others). Therefore, timely termination of NF-κB activation is crucial. Interestingly, some studies have shown that inhibition of cytokine signal 1 can degrade NF-κB and the PDLIM2 can act as a nuclear ubiquitin E3 ligase of p65, leading to p65 translocation to different subnuclear compartments for subsequent degradation by the 26 S proteasome (Tanaka et al., 2007). Furthermore, there is evidence to suggest that abnormal activation of NF-κB in osteoarthritis leads to joint inflammation and articular cartilage destruction. PDLIM2 can inhibit the activation of NF-κB signaling pathway, thereby alleviating LPS-induced chondrocyte injury (Guo et al., 2020; Saito and Tanaka, 2017).

Previous studies have also shown that the regulatory effect on NF-κB is reduced in PDLIM2 gene knockout mice, resulting in enhanced inflammation in mice with a high-fat diet, further leading to liver inflammation (Ya-Rong et al., 2018). There is also evidence in the literature showing that the PDLIM2 promotor is hypermethylated in tumors caused by Kaposi sarcoma herpesvirus, resulting in decreased expression of PDLIM2 and sustained NF-κB and STAT activation, leading to increased subsequent tumorigeneses (Sun et al., 2015). In addition, miR-222 can over activate the NF-κB signaling pathway in breast cancer cells by downregulating PDLIM2 expression, leading to increased tumorigenicity in breast cancer cells (Ding et al., 2018).

### 4.2 PDLIM2 and the TGF-β/Smad signaling pathway

**FIGURE 1:**
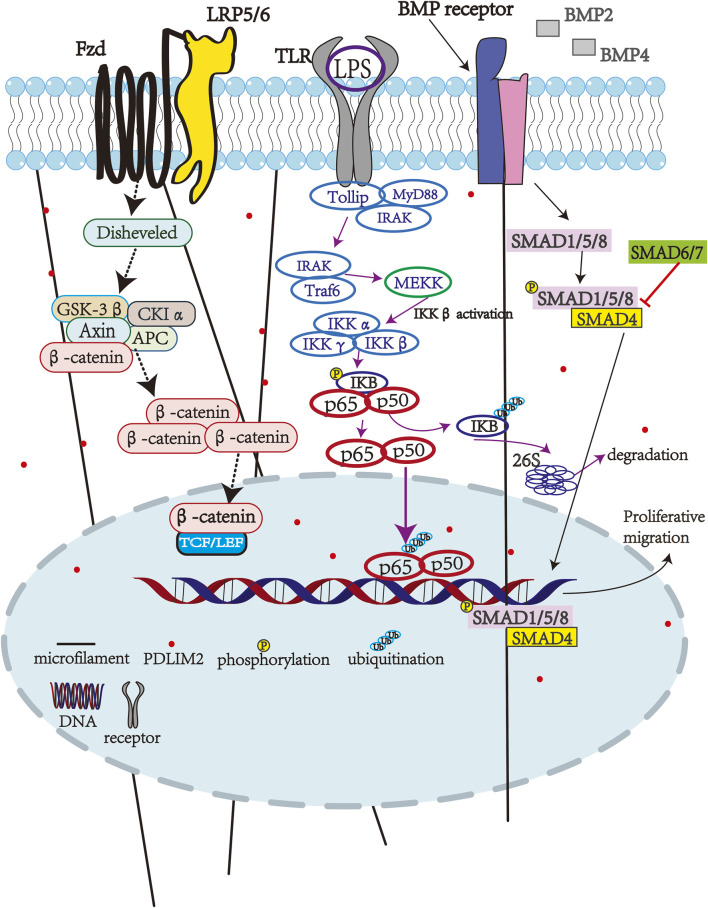
PDLIM2-associated pathways.

The TGF-β/Smad signaling pathway plays an important role in tumor cell proliferation, migration, and invasion. PDLIM2 expression is also downregulated in ovarian cancer tissues and cells leading to increased malignant biological behaviors (i.e., proliferation, migration, and invasion) (Lv et al., 2023). Importantly, PDLIM2 overexpression in ovarian cancer cells inactivates the TGF-β/Smad pathway to reduce these malignancies, thereby suggesting a role for this pathway in ovarian cancer pathogenesis. Indeed, *in vivo* mouse experiments have proven that upregulation of PDLIM2 can inhibit ovarian cancer cell proliferation. Therefore, PDLIM2 may also be a promising target for ovarian cancer treatment. In addition, PDLIM2 dysregulation is also closely related to several human cancers (e.g., hepatocellular carcinoma), and is associated with poor prognosis of patients, making it a potentially effective target for other cancer types as well (Figure 1) (Lv et al., 2023).

## 5 The role of PDLIM2 in tumorigenesis

Hepatitis B virus-induced hepatitis, cirrhosis, and liver cancer are thought to form a triad in liver cancer pathogenesis, thereby implying a potential correlation between inflammation and carcinogenesis (DiDonato et al., 2012). PDLIM2 expression is downregulated in steatohepatitis, and experimental evidence has demonstrated that deletion of the PDLIM2 gene promotes hepatic dyslipidemia and exacerbates insulin resistance in mice. Moreover, PDLIM2-knockout mice that were fed a high-fat diet showed increased pro-inflammatory cytokine secretion compared to wild-type mice under similar dietary conditions (Ya-Rong et al., 2018). These findings suggest that PDLIM2 serves as an effective inhibitor against steatohepatitis by potentially exerting inhibitory effects on NF-κB activity (Ya-Rong et al., 2018; Ji et al., 2024). Furthermore, inflammation-induced activation of NF-κB has been proven to facilitate tumor growth and metastasis. In addition, elevated expression levels of this protein have been associated with poor prognosis in certain cancers and tumors (GUO, 2005). Importantly, constitutive activation of NF-κB is implicated in the development of many cancers (DiDonato et al., 2012), and PDLIM2 expression has also been shown to be downregulated during this process, which raises the question: what is the connection between the two?

Compared with normal lung tissue, the expression of PDLIM2 in lung cancer is downregulated, which is related to the hypermethylation of the PDLIM2 promoter, treatment with DNA methyltransferase inhibitors can reverse the methylation of the PDLIM2 promoter, thereby restoring its expression in lung cancer cells (Sun et al., 2019). The low expression of PDLIM2 in lung cancer promotes the malignant progression of tumors, and the mechanism may be related to mitochondrial dysfunction and tumor metabolite accumulation caused by the increase of mitochondrial reactive oxygen species (ROS) (Yang J. X. et al., 2024). Additionally, PDLIM2 degrades STAT3 and the intranuclear transcription factor RelA (p65) through the ubiquitination-proteasome pathway, which exerts a cancer-inhibiting effect and thus inhibits the pathological process of lung cancer (Tanaka et al., 2007).

Therefore, maintaining normal expression levels of PDLIM2 is crucial for suppressing the onset and progression of lung cancer. Another mechanism involved in lung tumorigenesis is oxidative stress-induced translocation of the BTB domain and CNC homolog 1 (BACH1) protein into alveolar macrophage nuclei, where it binds to the PDLIM2 promoter, resulting in transcriptional inhibition of PDLIM2. The loss of PDLIM2 in alveolar macrophages leads to recruitment and transformation of mononuclear macrophages from blood circulation into lung macrophages, thereby promoting tumor activation within alveolar macrophages while impairing cytotoxic T lymphocyte-mediated phagocytosis, ultimately facilitating tumor development. This pathway involves the ROS-BACH1-PDLIM2-STAT3 signaling pathway (Li L. et al., 2021). Restoring PDLIM2 expression can also suppress p65 and STAT3 activity enhance the expression of genes involved in antigen presentation and T cell activation, and inhibit cancer-related genes, thereby rendering cancer cells more susceptible to immune attack (Sun et al., 2019). Similarly, PDLIM2 exhibits low expression in non-small cell lung cancer, leading to the restricted proliferation and invasion of non-small cell lung cancer (NSCLC) cells (Shi et al., 2020), as well as other malignant behaviors. This effect is mediated through PDLIM2-mediated degradation of p65, leading to subsequent inhibition of NF-κB transcriptional activity (Denlinger et al., 2004). Furthermore, the absence of PDLIM2 significantly augments the malignant behavior of NSCLC cells, and reduced expression levels are associated with poor patient survival in lung cancer (Baker et al., 2011).

Additionally, PDLIM2 is implicated in the pathogenesis of colon cancer. The constitutive activation of NF-κB plays a pivotal role in the development of intestinal diseases, and PDLIM2 can impede NF-κB activation. Epigenetic inhibition of PDLIM2 leads to reduced expression across various intestinal cancer cell lines, indicating that the loss of PDLIM2 is a significant contributor to NF-κB activation, with epigenetic inhibition being associated with promoter methylation (Qu et al., 2010b). Methylation-mediated gene silencing serves as a prevalent mechanism for inhibiting PDLIM2 in tumor tissues (Qu et al., 2010a; Cox et al., 2019). Overactivation of NF-κB leads to inflammatory diseases in humans. Importantly, chronic inflammation of the colon in the digestive system, particularly in Crohn’s disease and ulcerative colitis, is a major factor in colon cancer pathology. Previous studies have shown that treatment of colorectal cancer cell lines with DNA methyltransferase inhibitors rescues PDLIM2 expression, leading to inhibition of NF-κB activation in a dose-dependent manner and a subsequent reduction in tumor growth (Qu et al., 2010b). *In vivo* experiments in mice have also demonstrated that PDLIM2 can reduce the tumorigenicity of colorectal cancer cell lines, thereby providing a potentially promising therapeutic target for colorectal cancer drug discovery. In addition, PDLIM2-mediated inhibition of NF-κB activation has been shown to downregulate the release of inflammatory factors, including interleukin-1β (IL-1β), IL-6, and tumor necrosis factor alpha (TNF-α), thereby reducing lipopolysaccharide (LPS) -induced damage of articular chondrocytes (Guo et al., 2020). Thus, the anti-oncogenic activity of PDLIM2 lies in its inhibition of NF-κB activation.

PDLIM2 exhibits a dual role in cancer progression: it functions as a tumor suppressor in hormone-sensitive breast cancers and other malignancies, while paradoxically displaying elevated expression and pro-metastatic activity in triple-negative breast cancer (TNBC) and advanced metastatic tumors.

PDLIM2 is highly expressed in a subset of triple-negative breast cancer types, where it is only present in the cytoplasm and cell membranes, but not in the nucleus (Cox et al., 2019). In triple-negative breast cancer (TNBC), PDLIM2 is predominantly localized to the cytoplasm, and this spatial restriction positively correlates with β-catenin activation and adhesion signaling. In contrast, its nuclear translocation is mechanistically linked to the insulin-like growth factor-1 (IGF-1) and TGF-β pathways (Cox et al., 2019). Distant metastases of breast cancer cells is also the main cause of death in these patients. Thus, inhibiting metastases is an important clinical strategy in breast cancer treatment (Table 1). Importantly, vitamin D has anticancer activity, and 1,25-(OH)_2_ vitamin D3 is an activated form of vitamin D3, which inhibits tumor cell proliferation to slow down tumor growth. In breast cancer, the promoter fragment of PDLIM2 is hypermethylated, and 1,25-(OH)_2_ vitamin D3 can reduce the promoter methylation level to induce PDLIM2 expression. This induction is vitamin D receptor-dependent, thereby providing an additional biological target for future drug development strategies for breast cancer (Jemal et al., 2011).

**TABLE 1:** PDLIM2-associated tumor.

Cancer type	PDLIM2 role	Key mechanism
Metastatic kidney cancer	Promote	—
Prostate cancer	Promote	MAPK/ERK signaling
Meningioma、schwannoma	Promote	—
Breast cancer	Suppresses	NF-κB
Lung cancer	Suppresses	NF-κB, STAT
Colon cancer	Suppresses	NF-κB
Kaposi sarcoma	Suppresses	NF-κB、STAT
Ovarian cancer	Suppresses	TGF-β/Smad
Lymphoma	Suppresses	NF-κB

PDLIM2 plays a tumor suppressive role in breast cancer, colorectal cancer, lung cancer, lymphatic cancer, kidney renal papillary cell carcinoma (KIRP) and ovarian cancer (Sun et al., 2019; Denlinger et al., 2004; Zeng et al., 2022), while it is highly expressed in primary tumor tissues of metastatic kidney cancer, prostate cancer, meningioma, and schwannoma (Kang et al., 2016; Bassiri et al., 2017; Piao et al., 2022). The reason behind the differential expression of PDLIM2 in different caner types, the role that PDLIM2 plays in the pathogenesis of these cancers, and the mechanisms and pathways that are involved are all questions that require further investigation.

In addition to the aforementioned cancers, PDLIM2 has also been linked to infections of the central nervous system, and pathogenic microorganisms that infect the central nervous system can use PDLIM2 to cross the blood-brain barrier, PDLIM2 is also associated with vascular inflammation, rheumatoid arthritis, Laryngeal squamous cell carcinoma,and other diseases, which may present opportunities for additional novel strategies for PDLIM2-targeted therapies for these indications in the future (Table 2) (Li Z. et al., 2021; Wang S. et al., 2022; Yamamoto et al., 2023; Wang P. et al., 2022).

**TABLE 2:** PDLIM2-associated diseases.

Diseases	Associated processes	References
Metastatic kidney cancer	proliferation and metastasis	Yuk et al. (2021)
Breast cancer	progression	Cox et al. (2022), Qu et al. (2010b), Cox et al. (2022)
Lung cancer	progression	Li et al. (2021a), Shi et al. (2020), Kumar et al. (2018)
Colon cancer	progression	Qu et al. (2010b)
Articular chondrocyte injury	cell vitality	Guo et al. (2020)
Prostate cancer	growth and invasiveness	Kang et al. (2016)
Meningioma、schwannoma	proliferation	Bassiri et al. (2017)
Kaposi sarcoma	tumor occurrence and maintenance	Sun et al. (2015)
Ovarian cancer	Proliferation, migration, invasion	Lv et al. (2023)
Lymphoma	Proliferation	Wurster et al. (2017)
Central nervous system infection	Invasion	Li et al. (2021b)
Rheumatoid arthritis	Proliferation, migration, inflammatory response	Wang et al. (2022a)
Vascular Inflammation	Cellular inflammation	Yamamoto et al. (2023)

## 6 Limitations and future perspectives

Future research on PDLIM2 should aim to address the following questions.

1) Current research on PDLIM2 mainly focuses on tumors. What role does PDLIM2, as a cytoskeletal protein, play in other physiological systems or in the pathogenesis of diseases?
2) PDLIM2 is differentially expressed in different cancers. The reason for this phenomenon remains unclear, thus necessitating investigations into the underlying mechanisms behind this observation.
3) Regarding strategies used to inhibit PDLIM2 expression, only viral transfection has been reported to date, and no lead molecules have been identified to inhibit PDLIM2 expression. Therefore, additional research into PDLIM2 inhibitors is also critical.
4) Although PDLIM2 plays a crucial role in binding actin and has attracted a lot of research interest, there has been little research into its effects on the actin cytoskeleton or other cytoskeletons, such as the cytoskeleton-associated proteins that bind to bridge the connections to the cytoskeleton. It will be important to address the question of whether PDLIM2 affect the shape and position of the cytoskeleton during its involvement in cell biological functions.

## 7 Conclusion

In this review, we discuss the known functions of the PDLIM2 protein, its role in cellular and physiological processes, as well as its involvement in signaling pathways. As a connexin, PDLIM2 can bind to actin and α-actinin and plays an important role in various aspects of cell development. In addition, PDLIM2 plays different roles in the occurrence and development of different types of cancer, and further research on PDLIM2 is conducive to a better understanding of these diseases.

## Abbreviations

PDLIM, PDZ and LIM Domain; PDLIM2, PDZ and LIM Domain 2; NF-κB, Nucleus factor kappa-B; STAT, Signal transducer and activator of transcription; FAK, Focal adhesion kinase; ROCK, Rho-associated kinase; BACH1, Transcription factor BTB-CNC homolog 1; TGF-β, Transforming growth factor beta.
